# PASS: A scoring system to evaluate persistent kidney injury in critically ill ICU adult patients

**DOI:** 10.12688/f1000research.134459.3

**Published:** 2024-09-16

**Authors:** Dattatray Prabhu, Sonali Dattatray Prabhu, Chakrapani Mahabala, Mayoor V Prabhu

**Affiliations:** 1 Department of Anaesthesiology, Kasturba Medical College Mangalore, Manipal Academy of Higher Education, Manipal, Karnataka, 575001, India; 2 Department Of Radiodiagnosis, Kasturba Medical College Mangalore, Manipal Academy of Higher Education, Manipal, Karnataka, 575001, India; 3 Department of Medicine, Kasturba Medical College, Mangalore, Manipal Academy of Higher Education, Manipal, Karnataka, 575001, India; 4 Department of Nephrology, Kasturba Medical College, Mangalore, Manipal Academy of Higher Education, Manipal, Karnataka, 575001, India

**Keywords:** AKI, Sepsis, AKI recovery, renal Resistive index, Persistent AKI scoring system, Critically ill, RVI, creatinine

## Abstract

**Background:**

We evaluated if the course of recovery from sepsis-induced acute kidney injury (AKI) can be predicted using variables collected at admission.

**Methods:**

A total of 63 patients admitted for sepsis-induced AKI in our Mangalore ICU were evaluated and baseline demographic and clinical/laboratory parameters, including serum creatinine (SCr), base excess (BE), Plethysmographic Variability Index (PVI), Caval Index, R wave variability index (RVI), mean arterial pressure (MAP) and renal resistivity index (RI) using renal doppler and need for inotropes were assessed on admission. Patients were managed as per standard protocol. After six hours of fluid resuscitation, patients were classified as volume responders or non-responders. Re-assessment was done at 24 hours and 72 hours after admission. Primary outcome was persistent AKI after 72 hours. Secondary outcome was initiation of dialysis or death within 15 days of admission.

**Results:**

A total of 34 subjects recovered from AKI, of whom 32 patients were volume responders and 31 were non-responders. Response to fluid, MAP at admission and six hours, BE at admission, inotrope requirement, and PVI at admission did not correlate with recovery. Multiple logistic regression showed that SCr < 2.36 mg%, RVI > 14.45 and RI < 0.8 on admission correlated with recovery and they were evaluated further to model AKI recovery and develop PASS. PASS score = (SCr points × 5.4) + (RVI points × 4.0) + (RI points × 6.2). One point each was allotted if SCr was < 2.36, RVI was > 14.45 and RI was <0.8, and 0 otherwise. A score > 7.8 predicted recovery with a sensitivity of 79.4%, specificity of 72.4%, PPV 81.8%, NPV 76.7% and AuROC of 0.85.

**Conclusions:**

The PASS score can be used to identify salvageable cases of sepsis-AKI, guiding fluid resuscitation and aiding early referral from rural to tertiary care centers for better management.

## Introduction

Many patients admitted in intensive care unit (ICU), present with acute kidney injury (AKI) caused by ischemia, hypoxia or nephrotoxicity, resulting in rapidly declining glomerular filtration rate (GFR), leading to an increased risk of morbidity and mortality. The prevalence of AKI in critically ill ICU patients is high, particularly in those with sepsis (20-50% rate of prevalence). ^1^ Reversibility of AKI is influenced by the recovery response, which is in turn affected by the extent of renal damage and potential for renal cell regeneration. ^2^ Hence, for such patients, rapid restoration of circulation as well as optimal perfusion pressure is of paramount importance. Persistent AKI, exceeding 48 hours, is shown to significantly increase mortality. ^2^ The clinical definition of AKI sums it up as a rapid decrease of GFR, leading to retention of nitrogenous waste products. AKI is diagnosed and staged using the RIFLE (Risk, Injury, Failure, Loss of kidney function, and End-stage kidney disease) classification and AKIN (Acute Kidney Injury Network) criteria. ^1^^,^^3^ Severity and causes of AKI vary and have direct effects on mortality. ^1^^,^^3^ The RIFLE criteria are one of the most commonly used criteria to define and diagnose AKI, developed by the Acute Dialysis Quality Initiative (ADQI); they determine AKI severity on the basis of serum creatine (SCr), GFR, and urine output. ^4^

Despite best efforts, AKI does not resolve in many cases. ^5^ The major challenge to clinicians is in prognostication and patient counseling. Many studies have proposed different models for this purpose. Some studies have used numerous biomarkers for prompt detection and predicting severity of AKI and thereby categorizing into appropriate risk groups at risk for progressive renal decline, requirement of RRT, or death. ^5^^–^^7^ Biomarkers like interleukin-18 (IL-18), cystatin C (Cys C), neutrophil gelatinase-associated lipocalin (NGAL), kidney injury molecule-1 (KIM-1) and liver-type fatty acid binding protein (L-FABP) are being used for prediction of AKI and probable recovery. ^6^^–^^8^ Most of the tools to measure these biomarkers are expensive and not routinely available in all healthcare facilities.

Our objective was to use easily available parameters and variables to predict the course of the disease in AKI. Ryo Matsuura *et al.* have published a scoring system called PARI (Persistent AKI Risk Index) to predict low risk persistent AKI in critically ill adult patients. ^9^ However, PARI does not enable assessment of patients at admission to decide on resuscitation. Hence this study included variables routinely collected at admission to predict the course of AKI and guide fluid resuscitation sepsis AKI. ^9^^,^^10^

## Methods

### Study design, data source and participants

This prospective cross-sectional study commenced after institutional ethics committee approval (see Ethical considerations at the end of the manuscript). Patients admitted to ICU during 2019-2020 with were included in this study, after informed written consent from all patients or their next of kins were obtained. This study utilized non-random sampling, with inclusion criteria being age > 18 years and patients with AKI and septic shock admitted to the ICU. ^11^ Exclusion criteria included pregnant women, patients diagnosed with renal artery stenosis, end-stage renal disease (ESRD) or chronic kidney disease (CKD), patients < 18 years, patients taking angiotensin-converting enzyme inhibitor (ACEI) or non-steroidal anti-inflammatory drugs (NSAID), patients with cirrhosis with hepatorenal syndrome, cardiorenal syndrome and patients with AKI secondary to urinary tract obstruction (diagnosed on imaging). KIDGO criteria do not recommend gender specific definitions for AKI. RI and RVI do not have gender specific cut offs.

### Methodology

A total of 63 patients were included in over an eight-month period. We assessed parameters like serum creatinine (SCr), base excess (BE) in arterial blood gas (ABG) analysis, Plethysmographic Variability Index (PVI), Caval Index (CI), R wave variability in ECG, Mean arterial blood pressure (MAP), and renal resistive index (RI) using renal doppler screening on admission in patients presenting with sepsis. CI was calculated using IVC diameters obtained by trans-abdominal ultrasound using formula the formula (IVC maximum diameter during expiration − IVC minimum diameter during inspiration)/IVC maximum diameter × 100 and expressed as percentage. R wave variability represents the amplitude change in R waves on ECG calculated as (highest QRS amplitude – shortest QRS amplitude in one respiratory cycle)/mean QRS amplitude in same cycle × 100 and expressed as percentage.

After 6 of hours of fluid resuscitation as per standard guidelines, hemodynamic status and volume status of the patient was assessed. Patients were classified as ‘volume responders’ or ‘non-responders’ depending on hemodynamic stabilization using parameters like systolic blood pressure (SBP) and MAP. Hemodynamic stabilization is characterized by MAP exceeding 65 mmHg beyond 1 hour with no change in the rate of catecholamine infusion or fluid vascular loading. Re-assessment of all variables including estimation of SCr, CI, BE, R wave variability, MAP and RI, was done at 24 hours and 72 hours after admission.

The primary outcome was persistent AKI after 72 h. The secondary outcome was initiation of dialysis and death within 15 days of admission. AKI that resolves in 3 days of inclusion with conventional standard treatment in the ICU is called transient AKI. The recovery from AKI is characterized by SCr decreasing by 50% or absence of diuretics indicating normalization of urine output or both. Persistent AKI is characterized by persistently higher SCr or oliguria. ^6^^,^^12^

### Statistical analysis

The expression of percentages was done using categorical variables with means and standard deviations being expressed by continuous variables. Binary logistic regression was used to predict the AKI recovery using multiple variables as stated above, unadjusted and adjusted with noradrenaline and volume response and other variables. Based on the odds ratios (OR), a predictive equation was derived, and efficiency testing was performed with the use of a receiver operating characteristic curve (ROC) analysis. This curve’s coordinates, that yielded best sensitivity and specificity, were taken as a cut-off to develop a model (PASS score) for predicting recovery from AKI. In order to test the efficacy of the equation, sensitivity, specificity, positive predictive value (PPV), negative predictive value (NPV) and accuracy were considered. Additionally, the calculation of Kappa scores was done for the PASS score. Decision tree analysis was used for preparing the flow chart. All statistical analyses were performed using SPSS v.20 software. Our data showed no statistically significant difference in S creatinine, RI and R wave amplitude variation at admission between the genders. Hence gender specific subanalysis was not carried out.

## Results

A total of 63 subjects admitted to ICU with AKI were included as study participants. The following parameters were studied at admission and repeated at 6 hours: MAP, SCr, BE, PVI, CI, R wave variability, and RI. Table 1 shows the patients’ baseline characteristics; patients were managed with standard protocols for fluid resuscitation and other specific treatments. Overall, 32 patients showed volume response with respect to hemodynamic parameters and 31 patients were non-responders to fluid resuscitations. A total of 34 subjects recovered from AKI. 11 Non-responders and 4 volume responders were dialysed. 34 patients (53.9%) needed vasopressors to maintain MAP above 65 mmHg of which 22 were in Non responders and 12 were volume responders.

**Table 1.** Baseline characteristics of the patients.

Age (Years)	55.98±14.27
Sex ratio	Males (M): 40 Females (F): 23
MAP ADMISSION (mmHg)	63.57±4.88
RI atADMISSION	0.80±0.08
R wave Avariability at ADMISSION	15.09±2.05
CAVAL INDEX ON ADMISSION	54.53±12.54
BE ON ADMISSION (- mEq)	14.62±4.45
SCr ON ADMISSION	2.47±0.66
PVI ON ADMISSION	18.46±4.289
Gender (Male:Females)	Males 40; Females 23
Co-morbidities:	HTN: 34 (53.9%), DM: 21(33.4%)

Ventilated: 11 patients were ventilated (17.5%) of which 7 were Non responders and 4 were volume responders.

Multiple logistic regression analysis showed that response to fluid (seen as change in MAP at 6 hours); MAP, BE and PVI at admission and requirement of noradrenaline did not correlate with recovery of AKI ( Table 2). RI, RVI and SCr at admission correlated well with recovery from AKI, which was statistically significant. Cut-offs for these parameters were derived from the ROC curve analysis. Hence, these three parameters were evaluated further to develop the model for predicting recovery from AKI. Multiple logistic regression showed that creatinine < 2.36 mg%, R wave > 14.45 and RI < 0.8 at the time of admission were correlated with recovery from AKI with adjusted ORs of 5.447, 4.032 and 6.208 respectively ( Table 3). Based on this, the following formula was constructed in which SCr, RVI and RI were allotted points:

• SCr < 2.36 was allotted 1 point and values > 2.36 was allotted 0 points;
• R wave variability > 14.45 was allotted 1 point and values < 14.45 were allotted 0 points
• RI < 0.8 was allotted 1 point and RI > 0.8 was allotted 0 points.

**Table 2.** Logistic regression analysis for prediction of non-recovery from AKI. Acronyms: S.Cr - Serum creatinine, BE - Base excess in Arterial blood gas analysis, PVI - Plethysmographic variability index, MAP - Mean arterial blood pressure, RI - renal resistive index, CI - confidence interval.

	Unadjusted		Adjusted for nor adrenalin and volume response		Adjusted for all other variables	
	OR (95% CI)	P value	OR (95% CI)	P value	OR (95% CI)	P value
MAP difference at 6 hours	0.883 (0.738, 1.058)	0.177	0.878 (0.725,1.063)	0.181	0.681 (0.409,1.13232734)	0.14
MAP at admission	1.001 (0.904, 1.109)	0.982	1.055 (0.94,1.185)	0.36	0.849 (0.629,1.147)	0.286
RI at admission	184159.441 (78.714, 430858802.455)	**0.002**	63532.03 (19.266, 209500675.204)	**0.007**	19780.188 (1.413, 276815193.317)	**0.042**
R wave variability at admission	0.564 (0.39, 0.817)	**0.002**	0.562 (0.361,0.873)	**0.01**	0.476 (0.261,0.868)	**0.015**
Caval Index At admission	0.971 (0.932, 1.012)	0.16	0.994 (0.932,1.06)	0.851	1.027 (0.938,1.125)	0.564
BE at admission	0.906 (0.806, 1.019)	0.101	0.98 (0.844,1.137)	0.786	0.948 (0.765,1.175)	0.626
SCr at admission	3.908 (1.459, 10.471)	**0.007**	3.428 (1.198,9.807)	**0.022**	4.248 (1.145,15.763)	**0.031**
PVI at admission	0.879 (0.759, 1.017)	0.084	0.9 (0.762,1.064)	0.217	1.024 (0.806,1.3)	0.847

**Table 3.** Multivariate analysis showing p-value, adjusted odds ratio and 95% confidence interval (CI) for odds for variables.

	P	Adjusted odds ratio	95% CI for odds ratio	
			Lower	Upper
Creatinine > 2.36	0.010	5.447	1.494	19.859
R wave variability > 14.45	0.038	4.032	1.083	15.011
Resistive Index < 0.8	0.005	6.208	1.750	22.021

### Persistent AKI Scoring System (PASS) formula ( Table 4)

**Table 4.** Persistent AKI score-: points to be alloted for variables.

Parameter	Cut-off	Points
Creatinine	<2.36	5.4
	>2.36	0
R wave variability at admission	>14.45	4.0
	<14.45	0
Resistive index on admission	<0.8	6.2
	>0.8	0

$$\left[\mathrm{SCr}\;\text{points}\times 5.4\right]+\left[R\;\text{wave variability points}\times 4.0\right]+\left[\mathrm{RI}\;\text{points}\times 6.2\right]$$

A total score > 7.8 predicted recovery from AKI.

**Figure 1.**
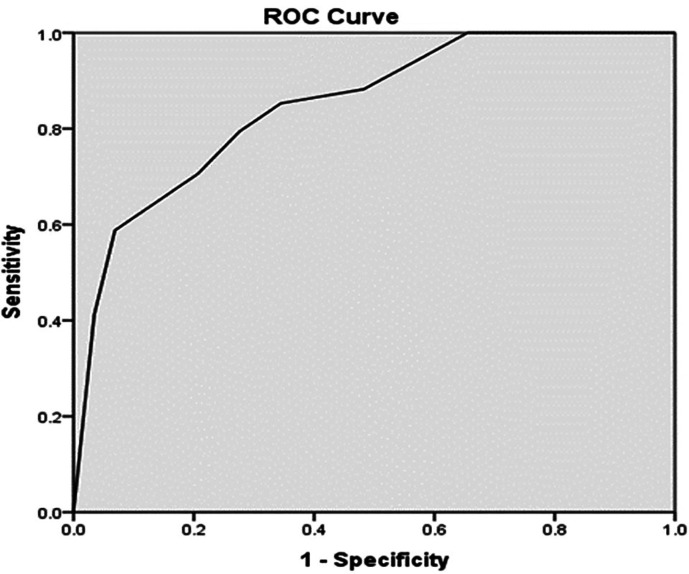
ROC curve for PASS score.

Sensitivity, specificity, predictive value, diagnostic accuracy and Kappa value (agreement with actual outcome) for SCr, RI at admission and RVI and for PASS score are shown in Table 5. A PASS Score > 7.8 had a sensitivity of 79.4% and 72.4% specificity for recovery from AKI. The PPV was 81.8% and NPV was 76.7%. The test and the gold standard agreed on 50 out of 63 having a diagnostic accuracy of 79.34%. The Kappa value of 0.586 indicated good agreement, with a p-value of < 0.001 ( Table 5). A PASS Score > 7.8, obtained by analysis of the ROC curve, had an ROC curve area of 0.85 (95% confidence interval of 0.755 to 0.943; p < 0.001) ( Figure 1).

**Table 5.** Sensitivity, specificity, predictive value, diagnostic accuracy and Kappa value forserum creatinine (SCr), Resistive Index (RI) at admission and R wave at admission, and of PASS study score.

Parameter	RI at admission < 0.8	SCr at admission < 2.36	R wave at admission > 14.45	PASS > 7.8
Sensitivity	79.30%	72.40%	73.50%	79.40%
Specificity	58.80%	70.60%	58.60%	79.30%
Positive predictive value	62.20%	67.70%	67.60%	81.80%
Negative predictive value	76.90%	75.00%	65.40%	76.70%
Diagnostic accuracy	68.25%	71.43%	66.67%	79.37%
Kappa statistics	0.374	0.428	0.324	0.586
p-value	0.004	0.001	0.012	<0.001

## Discussion

The Renal Angina Index, that detects minor variations in SCr along with other clinical variables, was proposed by some studies for identifying critically ill patients who are more likely to experience persistent AKI. ^12^^,^^13^ Few studies have tried to predict the severity of renal angina and utilized plasma and/or urinary biomarkers like cystatin C, L-FABP, NGAL, IL-18, KIM-1, among others, which are time consuming and expensive to measure. ^7^^,^^8^ In the Indian setting, some of the tools for measuring these biomarkers are not routinely available, limiting their widespread use. We have tried to utilize easily available data at the time of admission to develop a system that can solve this issue in developing countries. Some of these previous studies have used parameters at admission and after 24 hours for understanding whether AKI will be persistent. This method only helped to prognosticate patients after 24 h of admission. ^9^ As these test results are available after a lag time, they do not help clinicians in deciding at the time of admission whether patients will benefit from aggressive resuscitation. Thus, a robust system is needed, not only to assess but also to intervene at the earliest to reverse the damage.

**Figure 2.**
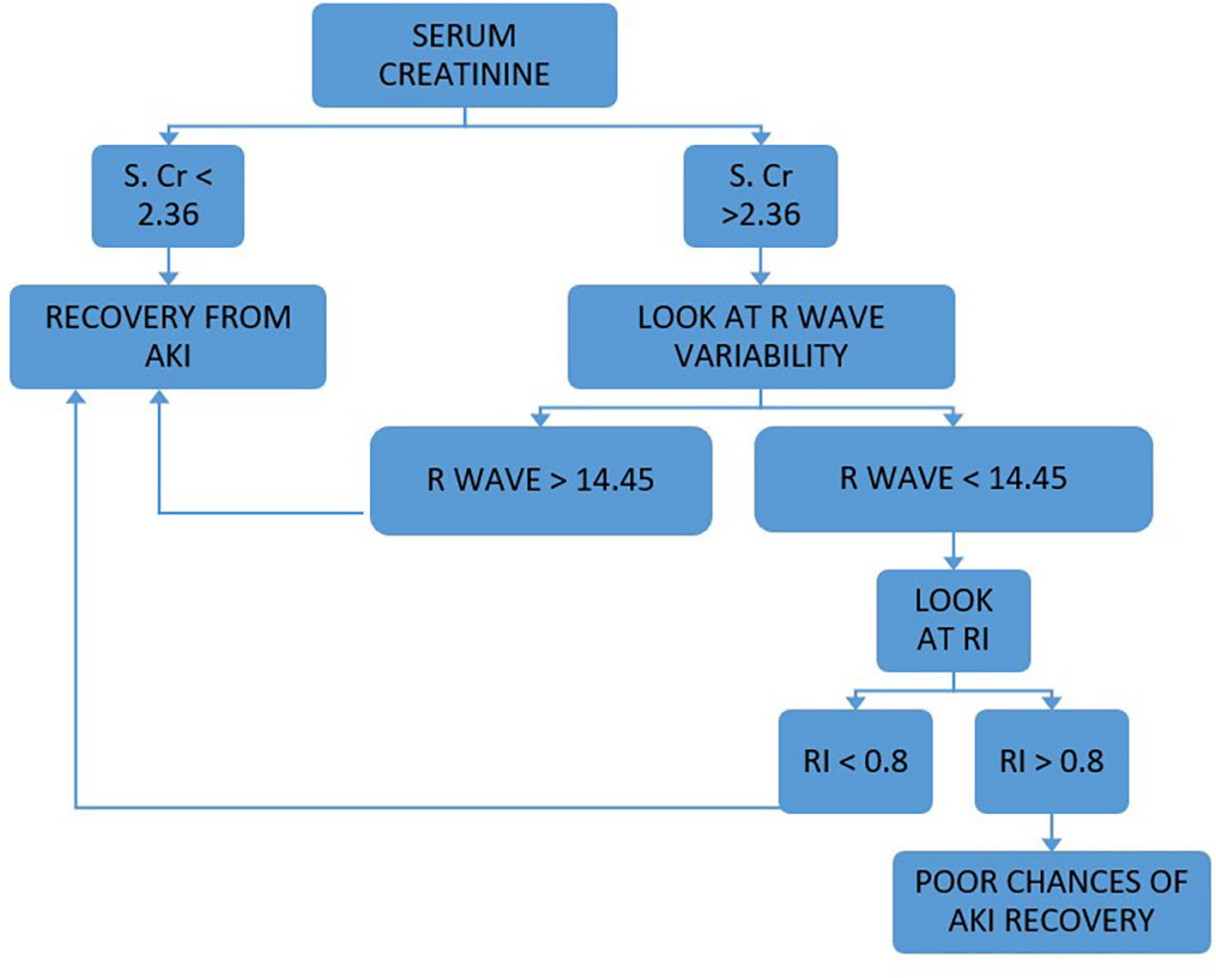
Flow chart for assessing renal anginal recovery i.e. recovery from AKI.

Our study concluded that SCr, RI and RVI at admission are statistically significant (p < 0.05) to predict AKI reversibility, which we have further analyzed to develop the PASS (Persistent AKI Scoring System) formula to predict the course of AKI. PASS is a combined scoring system that includes SCr levels, RI and RVI at admission. A total score greater than 7.8 predicted recovery from AKI. We also developed a flow chart for prediction of recovery from AKI which is shown in Figure 2. We found that the use of the PASS score and flow chart in adult ICU patients to be an effective tool to decide which patients have high risk of persistent AKI, and identify those who have potential for recovery from persistent AKI and will benefit from aggressive therapy, thus helping better prognostication.

Studies in the literature have also shown that RI is an important predictor of AKI and can even be used as a preoperative screening tool to predict AKI after surgeries. ^14^ Renal doppler and RI measurement can be done as a bedside tool to assess the renal circulation and are also a useful marker of sepsis ^14^; they can also help to differentiate persistent AKI from transient AKI in critically ill ICU patients. ^15^ RI is a non-invasive Doppler-measured parameter, corresponding to intra-renal arterial resistance and central hemodynamic parameters. Estimating increased RI on the first day of admission can help predict AKI in septic shock, and the literature has shown that these patients usually require mechanical ventilation. While some studies have shown higher RI values in AKI stages 2 and 3, this is not the case for patients in AKI stage 1. ^14^^,^^15^ A RI value beyond 0.795 predicts possibility of persistent AKI with good sensitivity and specificity. ^16^

Intravascular blood volume is dependent on the hemodynamic status of the patient and correlates with IVC diameter, in addition to morphological variations within the ECG like amplitude of waves. This phenomenon called “Brody effect”, related to the relationship of left ventricular volume on QRS-wave amplitude, can be identified by an increased amplitude of the QRS-wave due to increased ventricular preload. Thus, R wave variability is a reliable indicator for intravascular volume status variations. ^17^

Choudhary et al, in a large observation study using deep learning cluster analysis, have identified three distinct clusters in sepsis-induced AKI. The cluster with favourable outcome, in addition to having lesser severity of sepsis and also lesser multi organ involvement, had relatively lesser creatinine and lesser number of patients with creatinine criteria for AKI. Our study also confirmed that lower creatinine is associated with favourable outcome in sepsis induced AKI. Patients with sepsis-induced AKI had better come if they had only oliguric criteria as per Choudhary et al study. Our study also showed that patients with lower resistive index had favourable outcome indicating that patients without structural changes in kidney (as evidence by lower resistive index) had favourable outcome compared to those who had higher resistive index. ^18^

Our study is limited by being a single-centre study with a relatively smaller sample size. Wider application of our conclusions and predictive tool would require validation in larger multi-center studies.

## Conclusion

Acute kidney injury is very common in patients admitted to critical care. Invasive monitoring help us judge and guide resuscitation, but in rural areas facilities for these are not available. This study is to help these healthcare workers to use bed side non invasive parameters and blood reports at admission to assess and prognosticate kidney injury. Early identification of recoverable AKI patients will help in aggressive approach or early referral to tertiary center.

### Ethical considerations

Ethical approval was obtained from the Institutional Ethics Committee, Medical College, Mangaluru (Reg. No. ECR/541//nst/KA/2014/RR-17; Approval number IEC KMC MLR 08-19/327).

## Data Availability

Zenodo: PASS - A SCORING SYSTEM TO EVALUATE PERSISTENT ACUTE KIDNEY INJURY IN CRITICALLY ILL ADULT ICU PATIENTS, https://doi.org/10.5281/zenodo.7879938. ^19^ This project contains the following underlying data:
- PASS SCORE- AKI masterchart.xlsx Zenodo: PASS - A SCORING SYSTEM TO EVALUATE PERSISTENT ACUTE KIDNEY INJURY IN CRITICALLY ILL ADULT ICU PATIENTS, https://doi.org/10.5281/zenodo.7879938. ^19^ This project contains the following extended data:
- CHARTS AND TABLES (PASS SCORE-AKI).docx Data are available under the terms of the Creative Commons Attribution 4.0 International license (CC-BY 4.0).
