# Supplementary material for: PASS: A scoring system to evaluate persistent kidney injury in critically ill ICU adult patients
Source: F1000Res. 2024 Sep 16;12:902. Originally published 2023 Jul 28. [Version 3] doi: 10.12688/f1000research.134459.3 (PMC11733415; doi:10.12688/f1000research.134459.3)
Supplement: Peer review history

# Reviewer report: Reviewer response for version 3

**DOI:** 10.5256/f1000research.171311.r353011

**Published:** 2025-01-14

**Authors:** Dhruva Chaudhry

**Affiliations:** 1 Pt BD Sharma Post Graduate Institute of Medical Sciences, Rohtak, Haryana, India

Prabhu et al have tried to make an objective predictive model for resource limited countries or institutions. The article is well written ,however few corrections are required especially in Result section of Abstract.

 1. First line needs to be rewritten [Volume responders number are from total Cohort & not from patients recovered from AKI].

 2. The formula generated to calculate needs an explanation.

 3. As KIDGO is used, one does not find mention of Urine output? reason for exclusion of UO, may please be mentioned.

 4. Number in study is small (explanation given), strength seems to be prospective nature.

Is the work clearly and accurately presented and does it cite the current literature?

Yes

If applicable, is the statistical analysis and its interpretation appropriate?

Yes

Are all the source data underlying the results available to ensure full reproducibility?

Partly

Is the study design appropriate and is the work technically sound?

Yes

Are the conclusions drawn adequately supported by the results?

Yes

Are sufficient details of methods and analysis provided to allow replication by others?

Yes

Reviewer Expertise:

critical care,  respiratory medicine, public health, infectious diseases, policy etc.

I confirm that I have read this submission and believe that I have an appropriate level of expertise to confirm that it is of an acceptable scientific standard.

# Reviewer report: Reviewer response for version 2

**DOI:** 10.5256/f1000research.159669.r308310

**Published:** 2024-08-16

**Authors:** Anupam Agarwal

**Affiliations:** 1 The University of Alabama at Birmingham, Birmingham, Alabama, USA

The authors have provided a new scoring system - PASS - Persistent AKI Scoring System - to identify patients with sepsis-associated AKI who would need early referral to a tertiary care center.  Positive aspects of the study include the use of bedside non-invasive parameters and lab tests at admission to predict outcomes and early identification of patients who may need more aggressive management. Limitations are the small sample size of 63 patients which is duly acknowledged by the authors.

 Two points the authors should consider:

 1. Spell out the abbreviation PASS in the abstract under the results paragraph.

 2. Relate their findings in sepsis associated AKI and the use of the PASS scoring system to the work of Chaudhary et al (CJASN 2020, PMID: 33033164) who subphenotyped sepsis-associated AKI into three types based on deep learning and data from the electronic medical record.  This could be done in the discussion section of their paper.

Is the work clearly and accurately presented and does it cite the current literature?

Yes

If applicable, is the statistical analysis and its interpretation appropriate?

Yes

Are all the source data underlying the results available to ensure full reproducibility?

Yes

Is the study design appropriate and is the work technically sound?

Yes

Are the conclusions drawn adequately supported by the results?

Yes

Are sufficient details of methods and analysis provided to allow replication by others?

Yes

Reviewer Expertise:

Acute kidney injury

I confirm that I have read this submission and believe that I have an appropriate level of expertise to confirm that it is of an acceptable scientific standard.

# Reviewer report: Reviewer response for version 1

**DOI:** 10.5256/f1000research.147517.r211850

**Published:** 2023-10-24

**Authors:** Hernando Gómez

**Affiliations:** 1 University of Pittsburgh, Pittsburgh, Pennsylvania, USA

Title: PASS: A scoring system to evaluate persistent kidney injury in critically ill ICU adult patients

 Summary: Prabhu et al. present a prospective study in 63 patients with the objective to predict the course of sepsis induced AKI using easily available parameters and variables. The authors used serum creatinine (SCr), base excess (BE), Plethysmographic Variability Index (PVI), Caval Index, R wave variability index (RVI), mean arterial pressure (MAP) and renal resistivity index (RI) using renal doppler and need for inotropes were assessed on admission, 24 and 72h. They report that 34 patients recovered form AKI. Using multiple logistic regression, they determined thresholds and weights to compound the PASS score, ultimately composed of SCr, RVI and RI, achieving an AUC of 0.85, with sensitivity of 79.4%, specificity 72.4% for a threshold score of > 7.8 to predict recovery. The authors conclude that ‘the PASS score can be used to identify salvageable cases of sepsis-AKI, guiding fluid resuscitation and aiding early referral from rural to tertiary care centers for better management.’

 General comments: The manuscript is well written, and is focused on an important topic for the field. However, the enthusiasm is decreased due to significant methodological pitfalls. Specific examples and comments are detailed below.     

 Major comments:

1. The most important limitation of this study is the small sample size and the lack of validation of the PASS score. It is problematic to have less than 10 events per variable (EPV) when running this type of logistic regressions, as this can result in overfitting (type I error, PMID: 8970487). In the absence of a larger sample size and additional validation in an independent data set, it is difficult to consider the internal and external validity of the score.
2. Based on No. 1, it is difficult to accept the conclusion proposed by the authors. 
3. Based on the selection criteria, the PASS score may be applicable only to sepsis associated AKI, but not to other forms of AKI? Also, it is unclear what the timing of AKI diagnosis was in relation to sepsis, which is an important component of the definition of SA-AKI (PMID: 36823168)
4. The description of hemodynamic stabilization used a MAP of 60 mmHg instead of a MAP of 65 mmHg as recommended. Can the authors expand on the justification for the selection of this parameter?
5. The definition of volume responsiveness in the literature is not dependent on achieving a goal MAP or SBP, but rather on whether a bolus of fluid will increase cardiac output (or accepted surrogates) by at least 10%. The authors hereby use a different definition of volume responsiveness. The authors may consider modifying the terminology to align better with current definitions of volume responsiveness.
6. How did the authors define persistent AKI? The authors state they used SCr or oliguria to define persistent AKI. However, there is no definition of oliguria. Did the authors use KDIGO criteria?  
7. Was any stage AKI considered as persistent AKI if lasting > 72h?
8. How did the authors define transient AKI?
9. How did authors determine the presence of AKI? Was KDIGO staging used to defined AKI, and were both components (SCr and UO) used?
10. Please specify what were the covariates use to adjust the logistic regression model.
11. The description of the cohort is very limited, with only comporbidities shown including HTN and DM.

Minor comments

1. Please include measures of dispersion of the data in the form of standard deviation, IQR, etc.
2. Page 7. IVC diameter is not a reflection of intravascular volume. Rather, a specific threshold of static IVC diameter correlates with being above or below a certain CVP. Please revise this comment.
3. There is no conclusion in the manuscript. Please consider adding a conclusion.

Is the work clearly and accurately presented and does it cite the current literature?

Partly

If applicable, is the statistical analysis and its interpretation appropriate?

Partly

Are all the source data underlying the results available to ensure full reproducibility?

Yes

Is the study design appropriate and is the work technically sound?

No

Are the conclusions drawn adequately supported by the results?

No

Are sufficient details of methods and analysis provided to allow replication by others?

Partly

Reviewer Expertise:

Sepsis, AKI, metabolic reprogramming, organ dysfucntion

I confirm that I have read this submission and believe that I have an appropriate level of expertise to state that I do not consider it to be of an acceptable scientific standard, for reasons outlined above.

## References

[1] : A simulation study of the number of events per variable in logistic regression analysis. *J Clin Epidemiol* .1996;49(12) : 10.1016/s0895-4356(96)00236-3 1373-9 10.1016/s0895-4356(96)00236-3 8970487

[2] : Sepsis-associated acute kidney injury: consensus report of the 28th Acute Disease Quality Initiative workgroup. *Nat Rev Nephrol* .2023;19(6) : 10.1038/s41581-023-00683-3 401-417 10.1038/s41581-023-00683-3 36823168

# Response

**Published:** 2023-12-05

**Authors:** Dattatray Prabhu

**Affiliations:** anaesthesiology, Manipal Academy of Higher Education, Mangalore, Karnataka, India

*Dear Sir, I would like to thank you for taking the time to review this article.*

*This is a pragmatic trial as mentioned in the article , we accept its a small  sample size. Certain parameters among others had strong co-relation with AKI recovery. These parameters like R wave variability, Renal Resistive Index and initial S.Cr levels had a strong statistical significance to AKI recovery,so the conclusion was made. This needs a follow up study using larger study population for external validation.*

*Sepsis accounts for 50-70% of cases in our ICU. Sepsis-associated AKI  are usually clinical or subclinical at admission. This study includes sepsis related aki patients. During later part of ICU stay if AKI was found to be associated with non-sepsis-related causes such cases were excluded. The term SA-AKI operationally unifies the presence of AKI (according to clinical, biochemical and functional criteria) in the context of sepsis as a specific disease phenotype that is characterized by a specific trajectory and outcome.*

*The target  MAP for resuscitation was 65mmhg as per guideline. Correction was made in the manuscript accordingly. This study is mainly looking at non-invasive parameters to assess AKI recovery, hence we did not consider invasive monitors to look at cardiac output.*

*Recovery from AKI was attributed to decreasing creatine and normalizing urine output.*

*KIDGO criteria were used to categorize AKI after admission and recovery 24 hours after admission and beyond. Recovery of renal parameters and/or urine output beyond 72 hours were also considered as AKI recovery.*

*There are studies to show a strong correlation between IVC collapsibility and CVP. In this study, we have not considered CVP as it needs an invasive line and as previously mentioned this study is mainly focused on noninvasive parameters.*
